# Decoding Task-Based fMRI Data with Graph Neural Networks, Considering Individual Differences

**DOI:** 10.3390/brainsci12081094

**Published:** 2022-08-17

**Authors:** Maham Saeidi, Waldemar Karwowski, Farzad V. Farahani, Krzysztof Fiok, P. A. Hancock, Ben D. Sawyer, Leonardo Christov-Moore, Pamela K. Douglas

**Affiliations:** 1 Department of Industrial Engineering and Management Systems, University of Central Florida, Orlando, FL 32816, USA; 2 Department of Biostatistics, Johns Hopkins University, Baltimore, MD 21218, USA; 3 Department of Psychology, University of Central Florida, Orlando, FL 32816, USA; 4 Institute of Advanced Consciousness Studies, Santa Monica, CA 90403, USA; 5 School of Modeling, Simulation, and Training Computer Science, University of Central Florida, Orlando, FL 32816, USA

**Keywords:** task fMRI, brain decoding, classification, graph convolutional network, human connectome project

## Abstract

Task fMRI provides an opportunity to analyze the working mechanisms of the human brain during specific experimental paradigms. Deep learning models have increasingly been applied for decoding and encoding purposes study to representations in task fMRI data. More recently, graph neural networks, or neural networks models designed to leverage the properties of graph representations, have recently shown promise in task fMRI decoding studies. Here, we propose an end-to-end graph convolutional network (GCN) framework with three convolutional layers to classify task fMRI data from the Human Connectome Project dataset. We compared the predictive performance of our GCN model across four of the most widely used node embedding algorithms—NetMF, RandNE, Node2Vec, and Walklets—to automatically extract the structural properties of the nodes in the functional graph. The empirical results indicated that our GCN framework accurately predicted individual differences (0.978 and 0.976) with the NetMF and RandNE embedding methods, respectively. Furthermore, to assess the effects of individual differences, we tested the classification performance of the model on sub-datasets divided according to gender and fluid intelligence. Experimental results indicated significant differences in the classification predictions of gender, but not high/low fluid intelligence fMRI data. Our experiments yielded promising results and demonstrated the superior ability of our GCN in modeling task fMRI data.

## 1. Introduction

Functional magnetic resonance imaging (fMRI) is a non-invasive technology that provides high spatial resolution in determining the human brain’s responses [1]. Technically, fMRI estimates the regional brain activity by measuring metabolic changes in blood oxygen consumption associated with neural activity [2]. Modeling task fMRI data provides an opportunity to analyze the working mechanism of the human brain during performance of specific tasks. In task fMRI scanning, time series of the three-dimensional volume of the brain are acquired within a task block while the participant’s brain actively performs an explicit task. Pattern classification techniques are frequently applied to task fMRI data to determine if information is present in a particular brain region in a format the model can exploit, as in decoding studies. Encoding models can be used to make predictions about brain representations based on stimuli used in a particular task [3]. These models can also be applied to predict behavioral responses based on task fMRI [4], or for making group membership predictions based on graph theoretic features [5].

Graph neural networks (GNNs) have recently gained interest in the deep learning community, and have recently been applied to analyze task fMRI [6,7,8,9,10,11]. GNNs are neural network-based models that operate on graph-structured data, and have demonstrated state-of-the-art results in learning graph representations by embedding nodes in a low dimensional space [12]. Depending on the particular data structure of a graph, the performance of embedding methods in extracting the structural properties of graph nodes varies; thus, classification algorithms may perform differently according to the node embedding method used [13]. Although previous studies have attempted to increase the classification performance of task fMRI data by using various node embedding methods [8], less effort has been directed toward inferring how various node embeddings perform differently on classification task fMRI data. In this study, we used task fMRI data from the Human Connectome Project (HCP) dataset [14] to compare and adjudicate amongst embedding methods. Herein, we propose an end-to-end graph convolutional network (GCN) framework to classify task-evoked fMRI data. We conducted a series of experiments to evaluate the model’s classification performance by using four well-known node embedding algorithms: NetMF, RandNE, Node2Vec, and Walklets (Section 3.4).

Brain connectivity varies considerably amongst individuals [15,16,17,18,19], and even within individuals across time [20]. A number of studies have noted structural and functional connectivity differences across gender [21], developmental populations [5], intelligence [22], across the lifespan [23], and even level of empathic concern [24]. On the basis of such research indicating a close link between individual variability and brain connectivity derived measures, we aimed to examine the effects of individual differences (i.e., gender and fluid intelligence (gF)) on the classification performance of the proposed GCN model. For this purpose, we performed extensive experiments on four sub-datasets: gender-associated sub-datasets (female and male) and gF score-associated sub-datasets (sub-datasets associated with individuals with gF scores lower than the median value, denoted LM-gF, or higher than the median value, denoted HM-gF). We assessed significant changes in classification performance across each dataset. In general, our contributions through this work are summarized below:

- We propose an end-to-end GCN framework to classify task-evoked fMRI data. The objective is to examine the performance of various node embeddings to generate topological embeddings of the graph’s nodes. To our knowledge, this is the first investigation of different node embeddings on task fMRI classification performance. The code is available at https://github.com/krzysztoffiok/gnn-classification-pipeline, accessed on 20 February 2022.
- We demonstrate the performance of the proposed GCN framework according to individual differences (i.e., gender and fluid intelligence). To this end, we constructed four small sub-datasets of gender and gF score (LM-gF/HM-gF) with replacement.

This paper is organized as follows. In Section 2, we describe the background of the work. Section 3 describes our task fMRI data, the GCN architecture, and performance evaluation. In Section 4, we present the results from our experiments, followed by a discussion of our findings, the limitations of the work, and future directions. We end the current work with concluding remarks in Section 6.

## 2. Background

Over the past several decades, a variety of computational methods have been proposed to analyze fMRI time series data, such as the generalized linear model (GLM) [25,26], sparse dictionary learning [27,28,29,30], and blind source separation techniques including independent component analysis [31,32,33,34], non-negative matrix factorization [35], and tensor decomposition [36,37]. While useful, these techniques are either model free, or impose a particular inductive bias in the model. As such, their architectures do not resemble the structural or functional information processing in the human brain, limiting their capability of being used as brain computational models [38].

In the past several years, a growing body of literature has applied deep learning (DL) algorithms to fMRI data for decoding and encoding purposes. DL models leverage only a small subset of the dynamic capabilities of biological neurons, yet are functionally inspired by neurobiology. In DL methods, rather than using manual features, which are usually based on expert domain knowledge and heuristics [39], high level complex features can be automatically extracted from the original fMRI data, thus providing meaningful information to improve the performance of classification models. For example, Huang et al. [40] proposed a deep neural network model, consisting of both convolutional and recurrent layers, that automatically extracts spatial and temporal features of fMRI data. Their convolutional recurrent neural network model was used for the seven-class classification task, and the experimental results on the HCP dataset achieved an average accuracy performance of 94.3%. Wang et al. [41] applied a DL classifier with five convolutional layers and two fully connected layers on a large subset of task fMRI data from the HCP dataset and obtained an average accuracy of 93.7%.

Among DL models, convolutional autoencoders [42,43,44], recurrent autoencoders [45,46], and deep belief networks [47,48,49] have shown a superior ability to decode fMRI data. Huang et al. have developed a deep convolutional autoencoder to model fMRI data [42,43]; Zhao and colleagues used a spatio-temporal convolutional neural network to obtain both spatial and temporal features of functional networks [44]; Wang et al. have applied a deep sparse recurrent neural network on task fMRI data that has shown promising performance in extracting the temporal dependencies of input fMRI volumes [45]; and a deep belief network with a restricted Boltzmann machine [47] has been used to identify networks in fMRI data. Similarly, Jang et al. applied the deep belief network from [47] to initialize the weights of a fully connected deep learning architecture [48]. Despite the advances made by these methods, the DL models are yet to reach their full potential in the functional neuroimaging community due to the high dimensionality of the data and limited training data [50].

Graph-based network analyses capture information about the topological architecture of human brain networks [20]. Therefore, GNNs represent an attractive new tool for modeling brain information processing given that they are biologically inspired and leverage the hierarchical computing power from deep learning neural network models [51,52,53]. These models have been applied for fMRI decoding purposes using spectral-based GCN [6]. Li et al. [8] extended this work by proposing the BrainGNN framework with ROI-aware graph convolutional layers and ROI-selection pooling layers. These two types of layers were used to extract topological features of fMRI data and highlight the important nodes of the brain’s graph for prediction, respectively. The framework has been used to map regional and cross-regional functional activation patterns for decoding cognitive states in the HCP S1200 dataset. Furthermore, Kim et al. [10] considered the dynamic characteristics of the functional connectivity network and proposed the Spatio-Temporal Attention Graph Isomorphism Network for learning dynamic graph representation of the brain connectome with spatio-temporal attention.

## 3. Materials and Methods

According to the model used in [8], we applied a GCN framework for learning hierarchical representations of brain graphs to perform the node classification task. The topological and spatial feature vectors of brain functional graph nodes can also be automatically extracted by using node embedding methods. In this section, we first introduce the concept of convolutional operation on graph spectral domains, on the basis of Fourier transform and graph Laplacian. We then describe our proposed GCN model and the loss function that we intend to minimize. Finally, we present the dataset used as well as the brain network construction and feature extraction for graph nodes.

### 3.1. fMRI Dataset and Preprocessing

We obtained task fMRI data for 302 participants, consisting of 164 women and 138 men (22–35 years, mean =28.7±3.6) from the HCP 1200 Subject Release (S1200) [14]. HCP participants were randomly drawn from a population of healthy individuals, and fMRI data was collected while subjects performed seven different tasks—emotion, gambling, working memory, language, relational, social, and motor [26]. We used a subset of the HCP data collected at a single site, Washington University, to obviate the need for data harmonization [54]. Data were collected at 3 Tesla with TR = 0.72 s, TE = 33.1 msec, flip angle = 52 degree, FOV = 208 × 180 mm, and voxel size = 2.0 mm isotropic with opposite phase encoding directions (left-to-right and right-to-left). For further details, see [14].

To perform our experiments aimed at evaluation of the influence of individual differences, we considered two categories of task fMRI data: gender and fluid intelligence (gF). The first category consisted of two datasets in which task fMRI data for 164 and 138 participants were assigned to each sub-dataset according to gender. In the second category, we sorted gF scores of 302 participants in descending order and divided the dataset of 302 participants into two sub-datasets, LM-gF and HM-gF, of participants with gF scores lower than the median value (gF score <18) and with gF score higher than the median value (gF score ≥18), respectively. Consequently, a total of 144 and 158 participants’ task fMRI data were assigned to the LM-gF and HM-gF sub-datasets, respectively. Table 1 presents the demographics and participant distribution of the four defined sub-datasets.

The preprocessing of the task fMRI volume time-series was performed by the HCP consortium, as previously described [55]. The preprocessing pipeline included artifact removal and gradient distortion correction, motion correction, and registration to the standard Montreal Neurological Institute space with a DARTEL and voxel size of $2\times 2\times 2{\;\mathrm{mm}}^{3}$. Spatial smoothing and activation map generation were performed with a GLM implemented in FSL’s FILM (FMRIB’s Improved Linear Model with autocorrelation) [56]. More details regarding the HCP preprocessing pipeline can be found in Barch et al. [26].

### 3.2. Graph Convolutional Network: Spectral

#### 3.2.1. Notation

We used the basic notions described in [57]. A graph is defined as $G=\left(V,E\right)$ that consists of the set of nodes $\left\{v_{1},v_{2},\ldots ,v_{n}\right\}$ and set of edges such that $e_{ij}=\left(v_{i}\;,\;v_{j}\right)\in E$ and E⊆V×V. An edge e has two endpoints, $v_{i}$ and $v_{j}$, that are said to be joined by e. In this case, these two nodes are adjacent. A graph can be either directed or undirected. With an undirected graph, edges have no orientation. In contrast to undirected graphs, directed graphs are the set of nodes connected by edges that have a direction associated with them. Furthermore, a graph is a weighted graph if a weight is assigned to each edge. These weights quantify the degree of interaction between the nodes or the volume of exchange.

**Definition** **1** **(adjacency** **matrix).** *The adjacency matrix* A *for a graph* G *with* n *-nodes is an* n×n *matrix representation with* $A_{ij}=1$ *if direct connections exist between* $v_{i}$ *and* $v_{j}$ *, and* $A_{ij}=0$ *if no direct connections exist. If the graph is weighted, the entry of the adjacency matrix is* $A_{ij}>0$ *if* $\left(v_{i},{\;v}_{j}\right)\in E$ *and* $A_{ij}=0$ *, otherwise.*

**Definition** **2** **(feature** **matrix).** *The node feature matrix*$X\in R^{V\times d}$*, where*V*is the number of nodes in the graph, and*d*is the number of node features, is a matrix with*$x_{i}\in R^{d}$*representing the*d*-dimensional feature vector of the node*v*. Similarly, the edge feature matrix*$X^{e}\in R^{M\times p}$*is a matrix with*$X_{v_{i},v_{j}}^{e}\in R^{p}$*representing the*p*-dimensional feature vector of the edge*$e_{ij}$.

**Definition** **3** **(Laplacian** **matrix).** *The Laplacian matrix (or graph Laplacian)*$L\in R^{N\times N}$*is defined as*L=D−A*, where*D*is the degree matrix,*$D_{ij}=\overset{n}{\underset{j=1}{\sum }}A_{ij}$*, and*A*is the adjacency matrix of the unweighted graph. Similarly, for a weighted graph,*L=D−W, where W*is a weighted adjacent matrix. The symmetric normalized Laplacian matrix can be defined as*$L_{\mathrm{sym}}=I-D^{-\frac{1}{2}}{\mathrm{AD}}^{-\frac{1}{2}}$*, where*I*is the identity matrix.*

#### 3.2.2. Spectral-Based GCN

Spectral GCNs use the Laplacian matrix to compute the eigen-decomposition of the graph Laplacian in the Fourier domain. Let $L_{sym}$ be the symmetric normalized Laplacian matrix of graph $G.\;L_{sym}$ can be decomposed into $L_{sym}=U\Lambda U^{T}$, where $U=\left(U_{0},U_{1},\ldots ,U_{n-1}\right)\in R^{n\times n}$ is the eigenvector matrix, and Λ is the diagonal matrix of eigenvalues, $\Lambda =diag(\lambda_{1},\lambda_{2},\ldots ,\lambda_{n}).$ In graph signal processing, node features are mapped to feature vectors (i.e., $x_{0},,\ldots x_{n-1}$), which may be formed as a feature vector of all nodes of a graph, $X\in R^{n}$. The graph Fourier transform to a signal X is defined as $\hat{X}=U^{T}X$, and the inverse graph Fourier transform is defined as $X=U\hat{X}$. The graph convolution operation of X in the Fourier domain is defined as follows:

(1) $$X\times g=U\left(\left(U^{T}g\right)⊙\left(U^{T}X\right)\right)$$

where × represents convolution operation, ⊙ represents the pointwise product, and $g\in R^{N}$ represents the learnable parameters of the graph convolutional kernel. By defining $g_{\theta }=diag\left(U^{T}g\right)$ as a spectral filter in the spectral domain, the graph convolution operation can be simply defined as follows:

(2) $$X\times g_{\theta }=Ug_{\theta }\left(\Lambda \right)U^{T}X$$

Equation (2) was used for the first spectral network proposed [58]. However, this operation was computationally expensive because of multiplication eigenvector matrix U, which is a full matrix with n Fourier functions. To avoid the quadratic complexity, Defferrard et al. [59] have proposed ChebNet model, which avoids the eigen-decomposition by using a learning function of the Laplacian. The ChebNet model uses Chebyshev polynomials of the diagonal matrix of eigenvalues to estimate the filter $g_{\theta }$ as shown below:

(3) $$g_{\theta }=\overset{K}{\underset{i=0}{\sum }}\theta_{i}T_{k}(\tilde{\Lambda })$$

where $\tilde{\Lambda }=2\Lambda /\lambda_{max}-I_{N}$, and $\Lambda \in \left[-1,1\right]$. The model uses a Chebyshev polynomial for recursive calculation as $T_{k}\left(x\right)=2xT_{k-1}\left(x\right)-T_{k-2}\left(x\right)$ with $T_{0}\left(x\right)=1$ and $T_{1}\left(x\right)=x$. Therefore, the definition of the convolution of the graph signal x with a filter $g_{\theta }$ is as shown below:

(4) $$X\times g_{\theta }=U(\overset{K}{\underset{i=0}{\sum }}\theta_{i}T_{i}(\tilde{L}))U^{T}X$$

where $\tilde{L}=2L_{sym}/\lambda_{max}-I_{N}$, and maps the eigenvalues from $\left[0,\lambda_{max}\;\right]$ to $\left[-1,1\right]$ [60].

The filters defined by ChebNet are unstable for localizing frequency bands of interest, which are essentially the graph communities. To improve the above-mentioned ChebNet model and reduce the overfitting problem [61], Kipf and Welling [62] have proposed the CayleyNet model to capture narrow frequency bands by using Cayley polynomials. ChebNet assumes a linear function with respect to K=1 and $\lambda_{max}=2$, which results in a simplification of Equation (4) as shown below:

(5) $$X\times g_{\theta }=f({\tilde{D}}^{-\frac{1}{2}}\tilde{A}{\tilde{D}}^{-\frac{1}{2}}X\Theta )$$

where $\tilde{A}=I+A$, is an adjusted adjacency matrix A, ${\tilde{D}}_{ij}=\sum j{\tilde{A}}_{ij}$, f is the activation function, and Θ is a matrix of filter parameters.

### 3.3. Functional Graph

The raw task fMRI data were preprocessed through the HCP minimal preprocessing pipeline [55] and denoised by using ICA-FIX [63] to remove spatial artifacts and to perform motion correction. Furthermore, we used a large-scale multimodal brain atlas to parcellate the brain regions into 360 anatomical areas by using HCP Multi-Modal Parcellation, which is based on a combination of cortical architecture, function, connectivity, and topography [64]. By parcellation, we define regions of interest that represent graph nodes for brain network construction. Theoretically, the construction of a functional graph involves two steps. Herein, we first averaged the time series of all voxels in the region. Then we computed the functional connectivity between each pair of averaged time series of brain region through Pearson’s correlation coefficient. We used Fisher’s z transformation to normalized r values to improve the normality, and obtained a 360×360 symmetric matrix A (adjacency matrix) for each participant.

### 3.4. Feature Engineering and Node Embedding Algorithms

Features from averaged time series of brain regions were extracted by using Time Series Feature Extraction on basis of Scalable Hypothesis tests (tsfresh), an efficient and scalable feature extraction algorithm for time series based on a Python package [65]. The tsfresh algorithm integrates the components from the hypothesis tests with the feature significance testing on the basis of the FRESH algorithm [66]. Each generated feature vector is independently assessed to identify its significance for the given target by quantifying *p*-values and is further evaluated through the Benjamini–Yekutieli procedure [67] to decide which features to keep. The features extracted by tsfresh consist of both basic and advanced characteristics of the time series, and a complete list of features along with their mathematical descriptions can be found in reference [66]. We selected a minimum set of relevant statistical features to prepare feature representations for each node as follows: “absolute_sum_of_changes”, “benford_correlation”, “c3” (i.e., a measure of non-linearity in the time series), “cid_ce” (i.e., a measure of complexity in the time series), “longest_strike_above_mean”, “variance”, “standard deviation”, “skewness”, and “quantile” (i.e., 0.25 quantile).

In addition to the statistical features obtained through the tsfresh algorithm, node embeddings were applied to automatically extract node attributes in graphs. Node embedding algorithms project nodes into low-dimensional vectors, such that nodes with similar topological structures are in proximity in the embedding space [68]. We used the Python framework Karate Club [69], which consists of at least 30 graph mining algorithms, for node and graph embedding. We compared the performance of four state-of-the-art node embedding algorithms: Walklets [70] and Node2Vec [71], which use sampled random walks to make the node embeddings; NetMF [72], a factorization-based model; and the recently proposed RandNE [73], which is based on a Gaussian random projection approach with the default dimension ordering.

Walklets. In this method, instead of the random walk process used in DeepWalk [74], sample node neighborhoods are approximated by skipping over nodes in each short random walk. Then the set of results of multiple skip lengths is used to train the model [13].

Node2Vec. This method is a modification of DeepWalk introducing parameters p and q to smoothly interpolate between breadth-first sampling and depth-first sampling. Parameter p controls the likelihood of immediately revisiting a node in the walk, whereas parameter q allows the search to differentiate between “inward” and “outward” nodes. In Node2Vec, a vector representation of a node is computed on the basis of the second order random walks in the graph, and the core assumption is that Node2Vec’s sampling strategy is based on a mixture of breadth-first sampling and depth-first sampling suited for structural equivalence (i.e., similar roles of nodes) and homophily (i.e., network community), respectively [75].

NetMF. This method is a matrix factorization-based algorithm based on the connection between DeepWalk’s implicit matrix and graph Laplacians [73]. NetMF uses a small subset of nodes and extracts embedding vectors by approximating the proximity between nodes and the subset with the help of graph Laplacians [76].

RandNE. This method of iterative random projection network embedding preserves high order proximity between nodes by using a Gaussian random projection method while reducing the time complexity [73].

### 3.5. Proposed Model

#### 3.5.1. Modular Architecture

**Figure 1:**
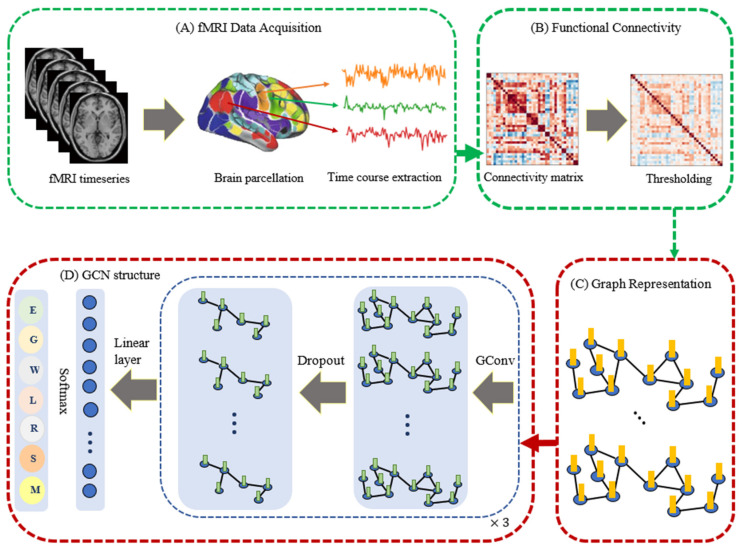
Overview of our Graph Convolutional Network (GCN) model for task fMRI classification. After acquisition of the raw task fMRI data and identification of the brain’s divisions into various parcels, several time courses of each parcel were extracted (**A**) to create the functional connectivity matrix. To reduce the complexity of the graph, a threshold was applied to the connectivity matrix (**B**) and transferred to a graph. The initial representation of each node was extracted by using the FRESH algorithm and node embedding methods (**C**). Finally, the feature vectors were used to perform the classification task with the proposed GCN framework including three Conv layers followed by a dropout layer after each Conv layer (**D**).

Our proposed model was developed by using PyTorch [77] and PyTorch Geometric [78]. The model takes a time series of fMRI volumes as input, in which each time series is a 2D matrix X of size T×N, where T is the number of time steps, and N is the number of brain regions. The tsfresh algorithm was used for statistical feature extraction for each node, and then high-level node features associated with each node were extracted with node embedding methods. The overall GCN model architecture for task fMRI classification is summarized in Figure 1.

The GCN model consists of three Conv layers with 92 neurons per layer. The Rectified Linear Unit (ReLU) and batch normalization layers are applied between each Conv layer to accelerate the convergence and enhance stability, and dropout layers are added after each Conv layer to reduce the inherent unnecessary complexity and redundant computation of our multilayer GCN model. Then a global mean pooling layer is applied to calculate the final graph representation vector. We performed experiments on the same computing machine equipped with a single NVIDIA Tesla T4 24 GB RAM GPU.

#### 3.5.2. Training and Testing

This study used five-fold stratified cross-validation within a training/validation/test setup. Four-fifths of the available data were allocated to a training set within each fold. The remaining one-fifth of the data were partitioned with a 60:40 ratio into a validation set and a final test set. The hyperparameter search consisted of a grid of learning rate, dropout, and weight decay values. The model with the lowest loss in the validation set was considered the best model for the final test. The following ideal parameters were used: learning rate: 0.001, dropout: 0.65, and weight decay: 0.0. Furthermore, because batch size is among the most important hyperparameters to tune, we considered a set of values of batch sizes. The batch sizes used in all experiments were $B=\left[16,\;32,\;48,\;64\right]$ over 100 epochs, all using the Adam optimizer and reducing the learning rate on a plateau with a patience of 10. Furthermore, cross-entropy loss was used for the optimization function.

#### 3.5.3. Evaluation Metrics

The metrics used for comparison embedding methods and evaluation of classification performance included accuracy, balanced accuracy, F1 scores (macro, micro, and weighted), Matthews correlation coefficient (MCC), precision, and recall. F1 macro and MCC have been widely considered as metrics to evaluate imbalanced datasets in which all classes are weighted equally [79,80]. Therefore, we applied accuracy, F1 macro, and MCC for further node embedding method comparisons and evaluation of GCN model performance. For statistical analysis, we used a significance threshold of 0.05. We also used the Shapiro–Wilk normality test [81] followed by the *t*-test to evaluate the statistical significance of the model’s classification performance in different scenarios.

## 4. Results

In this section, the experimental results are presented for the GCN model implementation and classification performance in different scenarios. Furthermore, the detailed information regarding the evaluation of node embeddings in the context of task fMRI decoding concerning gender and gF score differences is provided. Finally, we implemented classic univariate statistics to determine whether the difference in classification performance was statistically significant.

### 4.1. Classification of Task fMRI Data

The first set of results included the evaluation of our proposed GCN framework to classify which task the subject was performing during fMRI (7 classes) across node embedding techniques. The experiment was performed by using task fMRI data from the 302 participants, and the framework was set up by application of the four defined node embeddings regarding different batch sizes during training. The results are shown in Table 2. Table 2 illustrated that the RandNE and NetMF embedding methods outperformed the DeepWalk methods (Node2Vec and Walklets). This result might have been because DeepWalk-based methods require many sampled node neighborhoods to create node embedding vectors [82]. The F1 macro scores for RandNE and NetMF revealed similar performance across the GCN framework, and application of different batch sizes had a minor effect on the classification performance.

**Figure 2:**
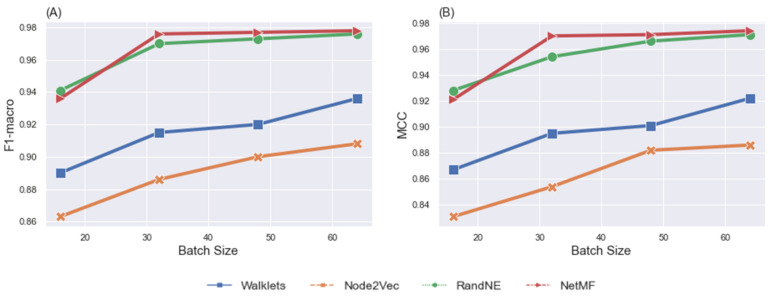
F1 macro (**A**) and MCC (**B**) comparison of the GCN model, taking into account the influence of node embedding methods and batch sizes for the task fMRI classification task, by using the 302 participants’ fMRI data.

**Figure 3:**
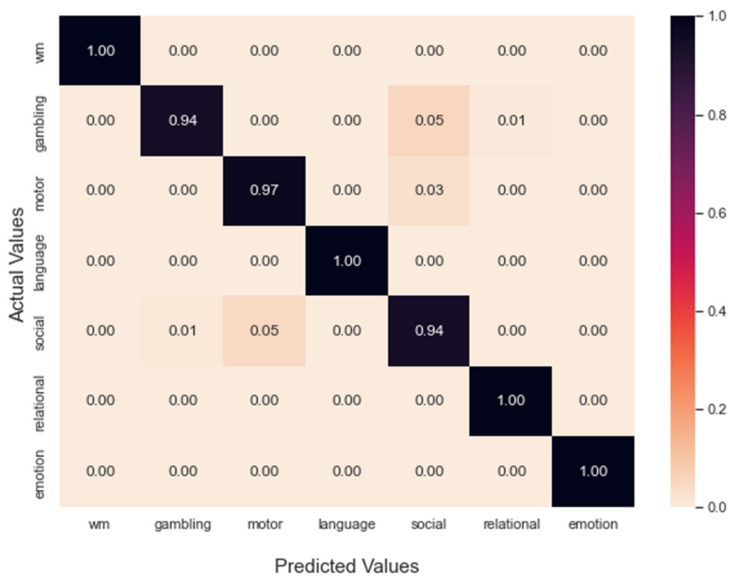
Confusion matrix of the GCN classification results on the 302 participants’ task fMRI data, normalized to the seven tasks in the five-fold cross-validation. The top two confusions were caused by the social task versus motor task and the gambling task versus social task. The F1 macro and MCC of classification were 0.977 and 0.974, respectively.

Figure 2 illustrates the effect of batch size on classification performance. As the number of batch sizes increased from 16 to 64, the F1 macro score and MCC increased. We also observed that using a batch size of 64 achieved superior results if any node embeddings were selected. However, our GCN model showed the best classification performance with NetMF when a batch size of 64 was chosen. We set up our GCN model and obtained the confusion matrix for task fMRI classification after the training step, as shown in Figure 3. The normalized confusion matrix indicated that the top confusions were between (1) the social and motor tasks and (2) the gambling and social tasks.

#### Performance Comparison

We compared the proposed GCN model with Logistic Regression (LR) that used L2 regularization, as our baseline model, to prove if the classification performance represented a noticeable improvement over the traditional machine learning model. LR works well as a baseline model since it is relatively easy to implement. The use of regularization prevents overfitting of the task fMRI data, so that the model features are shrunk towards zero and perform feature selection automatically. We evaluated the same brain decoding tasks and ran LR on our task fMRI dataset, splitting it into the train, validation, and test sets. To tune the regularization parameter, we used a range of values and perform a 5-fold cross-validation to achieve the optimal regularization parameter of 0.1. The result of L2-regularized LR showed a lower prediction accuracy in the seven-class classification task (97.7% vs. 86.4%, respectively, for GCN and LR with L2 regularization).

### 4.2. Effects of Group Membership on Classification

We performed experiments to evaluate the effects of gender and gF score on classification performance by using task fMRI data. The experiments were performed separately on the datasets described in Section 3.1. We applied the proposed GCN framework with the same hyperparameters above for all classification experiments.

#### 4.2.1. Gender Predictions

**Figure 4:**
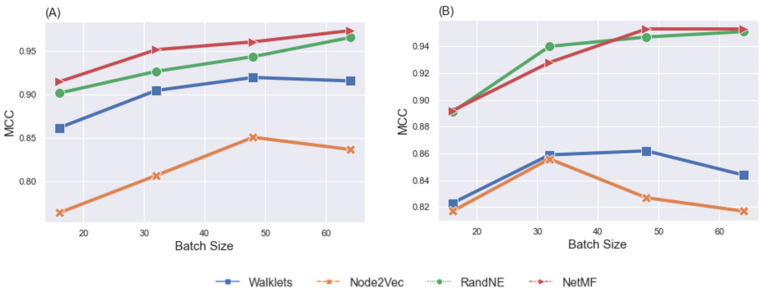
MCC comparison of the GCN model, taking into account the effects of node embedding methods and batch sizes, by using female task fMRI data (**A**) and male task fMRI data (**B**).

Classification. We first assessed the predictive performance of our model on predicting gender. The classification performance of the GCN model was evaluated across the four node embedding methods, and batch sizes were varied during training (Table 3). Several observations were made. First, we observed that the average F1 macro of the classifier on both sub-datasets ranged from 79.5% to 97.9%. Second, the GCN model achieved the best classification performance with NetMF for both sub-datasets. Third, the GCN model was sensitive to the choice of batch size, such that the best performance was obtained with a batch size of 64 for male and female sub-datasets. Similar trends were observed in the performance of the GCN model for MCC in Figure 4. For MCC, the model performance across both sub-datasets ranged from 82% to 97% with batch sizes of 48 and 64, respectively.

**Figure 5:**
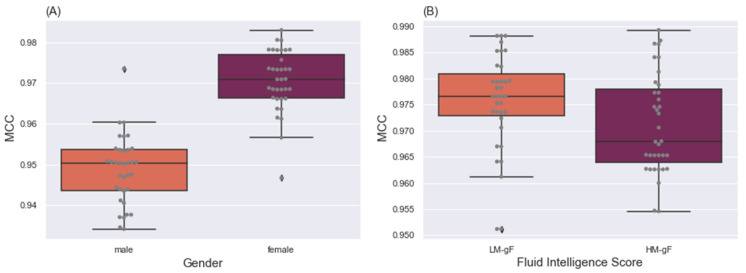
Box plots of the classification performance of the GCN model in 35 independent runs, by using gender sub-datasets (**A**) and fluid intelligence sub-datasets (**B**). Significant differences in classification performance of task fMRI data were observed between female and male data, but not between high and low fluid intelligence data.

Statistical analysis concerning MCC. According to the results of our model applied independently to female and male sub-datasets, the proposed GCN model had the best classification performance when NetMF was the node embedding method, and the batch size of 64 was selected during training. We set up the GCN model with NetMF and trained it with a batch size of 64, by using a learning rate of 0.001 for 100 epochs to classify task fMRI data for each sub-dataset separately. This process was performed iteratively a total of 35 times, and related MCC values were used to assess the statistical significance of the differences in classification performance. Figure 5A represents the results of this process. The GCN model performed relatively similarly, whereas each run showed varying performance between two sub-datasets.

To perform statistical significance testing, we used the Shapiro–Wilk normality test to assess normality. After assessing the statistical significance of the difference between classification performance of two sub-datasets (i.e., female and male), we performed a *t*-test, which indicated a significant difference (p<0.00001). The null hypothesis for this test was that the mean of classification performance for two sub-datasets was identical. Together, these results revealed that differences between the male and female task fMRI data were significant, such that classification was more accurate for of female than male task fMRI data.

#### 4.2.2. Fluid Intelligence Level Discrepancy

**Figure 6:**
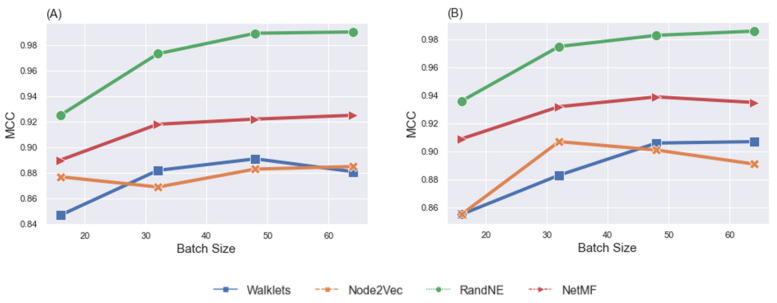
MCC comparison of the GCN model, taking into account the effects of node embedding methods and batch sizes, by using LM-gF task fMRI data (**A**) and HM-gF task fMRI data (**B**).

Classification. We evaluated the gF-score through the same procedures used for assessment of the influence of gender differences on classification task fMRI data. We set up the model and independently performed classification experiments on two sub-datasets: LM-gF and HM-gF. Table 4 shows the model’s performance regarding defined node embedding methods after training with a batch size range from 16 to 64 for LM-gF and HM-gF. Correspondingly, Figure 6 represents the visualization of the model’s performance for various node embeddings for MCC. The *x*-axis in the figures shows the batch sizes. The GCN classification showed high performance on LM-gF and HM-gF sub-datasets with RandNE and NetMF node embedding methods, which exhibited similar trends. In addition, the results indicated a change in performance of the model when the size of the batch increased from 16 to 64. The most striking observation was that for both sub-datasets, classification performance with RandNE achieved the best MCC.

Statistical analysis concerning MCC. To assess the influence of individuals’ gF-scores on the classification performance, we conducted the same procedure as the previous scenario for gender differences. However, we found that the GCN model had the best classification performance when RandNE was used as the node embedding method. Similarly, the model was set up and trained with a batch size of 48 and a learning rate of 0.001 for 100 epochs to classify task fMRI data by using LM-gF and HM-gF. We obtained two groups of values indicating the accuracy performance (i.e., MCC) of the classification model in different sub-datasets (Figure 5B). The Shapiro–Wilk normality test was performed to assess normality, and a *t*-test was used to assess the statistical significance of the differences in classification performance. The difference was found to be non-significant (p=0.604), at p<0.05. Therefore, the accuracy performance of classification task fMRI data for participants with lower and higher fluid intelligence was comparable. Thus, individuals’ gF scores do not affect task fMRI classification performance.

## 5. Discussion

### 5.1. Overview

In this study, we developed a GCN based model for classifying task fMRI data, or graph-structured data with associated nodal attributes. GCNs can aggregate higher-order information in “neighborhoods” from graph nodes representing regions of interest in the brain, and edges representing the functional connectivity [83,84,85,86]. Our study achieved an accuracy of 97.7% in a seven-class classification task, thus demonstrating a competitive classification performance for brain state decoding, with respect to those recently reported across task fMRI data by using the HCP dataset [8,40,41]. The comparisons of our multilayer GCN model with deep neural networks illustrate that node embedded features achieved better results than automatic feature extraction in DL. Our proposed method is clearly better than [40,41] that applied deep neural network models which obtained classification performances of 94.3% and 93.7%, respectively. Inspired by reference [8], our proposed model included three Conv layers, in which we first implemented several node embedding methods to extract the topological features of nodes and defined node weight. Then the first Conv layer was fed by using different node embedding weights instead of using the same weights for all nodes. To this end, we tested four node embeddings (i.e., NetMF, RandNE, Node2Vec, and Walklets) and observed that our GCN model using NetMF and RandNE tended to yield the best results for group membership classifying based on task fMRI data.

Furthermore, our findings confirmed the importance of selecting a proper node embedding method to extract topological features of graph nodes before feeding the GCN model, in agreement with previous research detecting influenza-like symptoms with a GNN model [82].

### 5.2. Effects of Individual Differences

We examined the effects of individual differences on task fMRI classification in terms of the gender and gF score discrepancy. With respect to gender differences, the performance of the proposed GCN model was tested on two sub-datasets (female/male) by considering four node embedding methods. The same procedure was applied on gF-associated sub-datasets (LM-gF/HM-gF). We observed a significant difference in task fMRI classification performance between gender sub-datasets. However, no significant difference was observed in classification performance at a 95% confidence interval, because the *p*-value was greater than 0.05.

### 5.3. Effects of Batch Size

Training a DL model involves selecting a large set of hyperparameters, among which batch size is important [87]. Batch size defines the number of training samples used in one iteration to update the internal network parameters. To achieve the best accuracy performance of the GCN model associated with the batch size values, we chose a sequence of batch sizes of 16, 32, 48, and 64, and applied GCN architectures to each dataset. This approach allowed us to obtain the best classification performance for each experiment. The trend of the batch size change influenced the classification performance for all considered datasets. The worst classification performance values were obtained with a batch size of 16, and the best results were achieved with batch sizes of 48 and 64.

### 5.4. Limitations and Future Work

Our current study has several limitations that should be considered in future research. Although this study examined several node embedding methods to represent the graph nodes as low dimensional vectors, we disregarded the influence of the dimensionality of the node embeddings. Although finding the optimal dimension for embedding methods is challenging, some studies have applied several embedding dimensions on various datasets and achieved varying performance [13,88]. Therefore, the node embedding method must be customized to our dataset in future work. Furthermore, although batch size is an important hyperparameter to be considered in training a DL model [87], and we refitted our GCN model with different batch sizes and analyzed the effects of the change in batch size on classification performance, more hyperparameters should be studied, such as the number of convolutional layers, pooling ratio, and different readout operations. Finally, we analyzed only the task fMRI dataset for 302 participants and concluded that gender differences can affect classification performance. However, the ability to generalize our findings should be studied over a large number of participants and evaluated using our decoding model for experimental conditions under each task fMRI.

## 6. Conclusions

We proposed a GCN model to decode task fMRI data from the HCP dataset. Four node embedding methods—NetMF, RandNE, Node2Vec, and Walklets—were used to extract the topological features of graph nodes. We compared the performance of the model with different node embeddings through experiments and assessed classification accuracy. Our GCN model not only performs better on classification than alternative methods but also offered a relatively simple GCN architecture in which dropout layers reduced the redundant computation of the model. We further examined whether individual differences affect task fMRI data classification performance. Several conclusions were drawn. First, the overall task fMRI classification of the GCN model resulted in an accuracy, F1 macro and MCC of 0.977, 0.978 and 0.974, respectively. Second, the most robust node embedding methods for task fMRI data were NetMF and RandNE, whereas the least robust node embedding method was Node2Vec. Third, the influence of gender differences on task fMRI classification performance was significant, whereas no significant difference was observed between gF score categories.

In general, the method of this study provides a robust graph neural network-based data analysis method and examined various node embedding methods to provide a more effective solution for analyzing task fMRI data. Developing methods to test and validate saliency methods used for explainable artificial intelligence is still an active area of research [89]. However, as these techniques mature, GCNs may represent an important new tool for modeling brain information processing, using architectures inspired by the structural and functional graph properties of the brain.

## Figures and Tables

**Table 1:** Demographics and participant distribution results.

Groups	Number of Participants	Age (Mean ± SD)	gF-Score (Mean ± SD)
Female	164	29.2 ± 3.6	-
Male	138	28.1 ± 3.6	-
LM-gF	F=91, M=53	29.1 ± 3.6	12.5 ± 3.4
HM-gF	F=83, M=75	28.4 ± 3.6	20.6 ± 1.7

Abbreviations: LM-gF, low median-gF score; HM-gF, high median-gF score; F, female; M, male.

**Table 2:** Two-factor performance comparison on predicting experimental task, taking into account the influence of node embedding methods and batch sizes for the task fMRI classification. The training processes were set with 100 epochs, 10 step patience for early stopping, and learning rate = 0.001 for Adam. The proposed GCN model showed impressive results with both RandNE and NetMF node embedding methods. Classification performance values for 302 participants’ task fMRI data were in the range of 94% to 98%. Bold values represent the best classification performance obtained for each batch size.

Batch Size	Node Embeddings	Metrics		
		Accuracy	F1 Macro	MCC
16	Walklets	0.886	0.89	0.867
	Node2Vec	0.854	0.863	0.831
	RandNE	**0.939**	**0.941**	**0.928**
	NetMF	0.933	0.936	0.921
32	Walklets	0.911	0.915	0.895
	Node2Vec	0.873	0.886	0.854
	RandNE	0.969	0.97	0.954
	NetMF	**0.974**	**0.976**	**0.97**
48	Walklets	0.915	0.92	0.901
	Node2Vec	0.898	0.9	0.882
	RandNE	0.971	0.973	0.966
	NetMF	**0.976**	**0.977**	**0.971**
64	Walklets	0.932	0.936	0.922
	Node2Vec	0.902	0.908	0.886
	RandNE	0.975	0.976	0.971
	NetMF	**0.977**	**0.978**	**0.974**

**Table 3:** Two-factor performance comparison, taking into account the influence of node embedding methods and batch sizes in the GCN model, by using both the female and the male fMRI data. Bold values represent the best classification performance obtained for each batch size.

Batch Size	Node Embeddings	Female Dataset			Male Dataset		
		Metrics			Metrics		
		Accuracy	F1 Macro	MCC	Accuracy	F1 Macro	MCC
16	Walklets	0.881	0.882	0.862	0.849	0.85	0.823
	Node2Vec	0.792	0.795	0.764	0.841	0.845	0.817
	RandNE	0.916	0.916	0.902	0.907	0.909	0.891
	NetMF	**0.927**	**0.928**	**0.915**	**0.908**	**0.911**	**0.892**
32	Walklets	0.919	0.919	0.905	0.879	0.882	0.859
	Node2Vec	0.835	0.837	0.807	0.878	0.879	0.856
	RandNE	0.938	0.939	0.927	**0.949**	**0.951**	**0.94**
	NetMF	**0.959**	**0.959**	**0.952**	0.939	0.941	0.928
48	Walklets	0.931	0.932	0.92	0.887	0.889	0.862
	Node2Vec	0.871	0.869	0.851	0.852	0.857	0.827
	RandNE	0.952	0.952	0.944	0.955	0.957	0.947
	NetMF	**0.967**	**0.967**	**0.961**	**0.962**	**0.964**	**0.953**
64	Walklets	0.928	0.928	0.916	0.871	0.874	0.844
	Node2Vec	0.859	0.861	0.837	0.845	0.849	0.817
	RandNE	0.971	0.972	0.966	0.958	0.961	0.951
	NetMF	**0.979**	**0.979**	**0.974**	**0.962**	**0.965**	**0.953**

**Table 4:** Two-factor performance comparisons, taking into account the influence of node embedding methods and batch sizes in the GCN model, by using both LM-gF and HM-gF task fMRI data. Bold values represent the best classification performance obtained for each batch size.

Batch Size	Node Embeddings	LM-gF Dataset			HM-gF Dataset		
		Metrics			Metrics		
		Accuracy	F1 Macro	MCC	Accuracy	F1 Macro	MCC
16	Walklets	0.869	0.873	0.847	0.876	0.876	0.855
	Node2Vec	0.895	0.896	0.877	0.876	0.878	0.855
	RandNE	**0.936**	**0.937**	**0.925**	**0.945**	**0.944**	**0.936**
	NetMF	0.906	0.908	0.89	0.921	0.921	0.909
32	Walklets	0.899	0.902	0.882	0.901	0.901	0.883
	Node2Vec	0.891	0.893	0.869	0.92	0.92	0.907
	RandNE	**0.977**	**0.977**	**0.973**	**0.98**	**0.979**	**0.975**
	NetMF	0.93	0.93	0.918	0.942	0.942	0.932
48	Walklets	0.908	0.91	0.891	0.92	0.919	0.906
	Node2Vec	0.9	0.902	0.883	0.915	0.915	0.901
	RandNE	**0.991**	**0.991**	**0.989**	**0.988**	**0.988**	**0.983**
	NetMF	0.934	0.934	0.922	0.948	0.947	0.939
64	Walklets	0.899	0.901	0.881	0.92	0.918	0.907
	Node2Vec	0.901	0.903	0.885	0.906	0.905	0.891
	RandNE	**0.991**	**0.991**	**0.99**	**0.988**	**0.988**	**0.986**
	NetMF	0.936	0.938	0.925	0.944	0.943	0.935

## Data Availability

The data presented in this study are available on request from the corresponding author.
